# Photonic crystal for graphene plasmons

**DOI:** 10.1038/s41467-019-12778-2

**Published:** 2019-10-21

**Authors:** L. Xiong, C. Forsythe, M. Jung, A. S. McLeod, S. S. Sunku, Y. M. Shao, G. X. Ni, A. J. Sternbach, S. Liu, J. H. Edgar, E. J. Mele, M. M. Fogler, G. Shvets, C. R. Dean, D. N. Basov

**Affiliations:** 1 Department of Physics, Columbia University, New York, NY 10027 USA; 2 Department of Physics, Cornell University, Ithaca, NY 14853 USA; 3 Department of Applied Physics and Applied Mathematics, Columbia University, New York, NY 10027 USA; 4 The Tim Taylor Department of Chemical Engineering, Kansas State University, Manhattan, KS 66506 USA; 5 Department of Physics and Astronomy, University of Pennsylvania, Philadelphia, PA 19104 USA; 6 Department of physics, University of California San Diego, La Jolla, CA 92093 USA; 7 School of Applied and Engineering Physics, Cornell University, Ithaca, NY 14853 USA

**Keywords:** Photonic crystals, Optical properties and devices, Optical properties and devices, Photonic crystals, Polaritons

## Abstract

Photonic crystals are commonly implemented in media with periodically varying optical properties. Photonic crystals enable exquisite control of light propagation in integrated optical circuits, and also emulate advanced physical concepts. However, common photonic crystals are unfit for in-operando on/off controls. We overcome this limitation and demonstrate a broadly tunable two-dimensional photonic crystal for surface plasmon polaritons. Our platform consists of a continuous graphene monolayer integrated in a back-gated platform with nano-structured gate insulators. Infrared nano-imaging reveals the formation of a photonic bandgap and strong modulation of the local plasmonic density of states that can be turned on/off or gradually tuned by the applied gate voltage. We also implement an artificial domain wall which supports highly confined one-dimensional plasmonic modes. Our electrostatically-tunable photonic crystals are derived from standard metal oxide semiconductor field effect transistor technology and pave a way for practical on-chip light manipulation.

## Abstract (web summary)

Traditional photonic crystals consist of periodic media with a pre-defined optical response. Here, the authors combine nanostructured back-gate insulators with a continuous layer of graphene to demonstrate an electrically tunable two-dimensional photonic crystal suitable for controlling the propagation of surface plasmon polaritons.

## Introduction

When light propagates in media with periodically varying optical properties, a collection of optical phenomena emerges which can be described under the notion of photonic crystal^1^. Photonic crystals support intriguing states of matter, including topological photonic states^2–5^, unidirectional edge modes^6,7^ and pseudo-magnetic fields for photons^8^. To date, photonic crystals have been fabricated exclusively in discrete media with built-in local electrodynamics responses^9–11^. Because the photonic crystal effect cannot be readily turned off, conventional systems typically have very limited tunability^9^.

A promising alternative platform for tunable photonic crystals can be constructed using two-dimensional (2D) van der Waals heterostructures. Unlike conventional materials, van der Waals heterostructures are widely tunable by a variety of readily attainable stimuli, including field-effect, optical pumping^12^, and mechanical pressure^13^. Specifically, in monolayer graphene heterostructures, electrostatic gating can be used to greatly modify the optical properties^14,15^. Under the illumination of infrared (IR) radiation, graphene heterostructures support low-loss surface plasmon polaritons (SPPs) modes that are hybrid excitations of IR photons and Dirac electrons^14,16–19^. At a laser frequency *ω*, the wavelength *λ*_p_ of graphene SPPs scales with the carrier density *n*_s_ as $\lambda _{\mathrm{p}} \propto \frac{{v_{\mathrm{F}}\sqrt {n_{\mathrm{s}}} }}{{{\it{\epsilon }}\omega ^2}}$, where $v_{\mathrm{F}} = 10^6\;{\mathrm{m}}\;{\mathrm{s}}^{ - 1}$ is the Fermi velocity and *ε* is the dielectric constant for the environment surrounding the graphene layer. By varying *n*_s_ through field effect, the propagation of SPP waves in graphene can be readily controlled^12,20–25^, making monolayer graphene heterostructures a suitable platform for implementing tunable photonic crystals.

Here, we propose, design and fabricate a tunable photonic crystal for SPPs out of a continuous graphene monolayer. The nanoscale spatial modulation of the graphene optical response is controlled by patterning a periodic structure in the SiO_2_ gate insulator layer^26^. Near-field imaging techniques reveal the formation of a complete plasmonic bandgap, in which SPPs propagation is prohibited. In the vicinity of the bandgap, plasmonic density of states shows strong spatial modulations and can be turned on/off and tuned by the applied gate voltage. An artificially designed domain wall in the photonic crystal supports highly confined one-dimensional (1D) plasmonic mode. Our graphene photonic crystal platform provides highly desired tunability for SPPs propagation, and is designed for on-chip in-operando light manipulation.

## Results

### Modeling of graphene SPP band structure

**Fig. 1:**
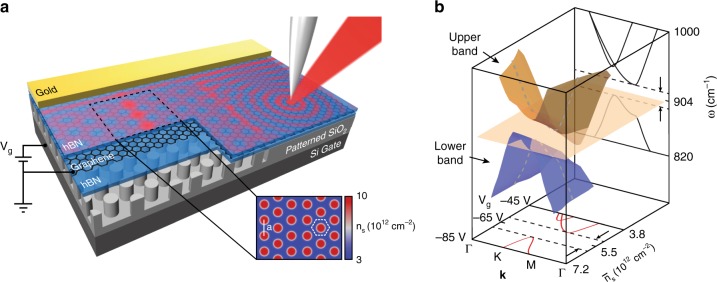
Graphene photonic crystal. **a** Schematic of a photonic crystal comprised of a graphene monolayer fully-encapsulated by hexagonal boron nitride on top of an array of SiO_2_ pillars. Inset, simulated carrier density map *n*_s_(**r**) at average carrier density $\bar n_{\mathrm{s}} = 4.5 \times 10^{12}\;{\mathrm{cm}}^{ - 2}$, showing two hexagonal-patterned domains separated by an artificial lattice dislocation (domain wall). The lattice periodicity *a* = 80 nm. The photonic crystal unit cell is marked by a white dashed hexagon. **b** Calculated plasmonic band structure as a function of wave-vector **k** and average carrier density $\bar n_{\mathrm{s}}$. A vertical cut parallel to the $\omega - {\mathbf{k}}$ plane (back panel) generates the plasmonic band structure at fixed carrier density $\bar n_{\mathrm{s}} = 5.5 \times 10^{12}\;{\mathrm{cm}}^{ - 2}$. The dashed lines mark the range of a complete plasmonic bandgap. A horizontal cut parallel to $\bar n_{\mathrm{s}} - {\mathbf{k}}$ plane (bottom panel) generates the plasmonic dispersion as a function of average carrier density $\bar n_{\mathrm{s}}$ and wave-vector **k**, at laser frequency *ω* = 904 cm^−1^; a complete bandgap is evident for carrier density around $\bar n_{\mathrm{s}} = 5.5 \times 10^{12}\;{\mathrm{cm}}^{ - 2}$

**Fig. 2:**
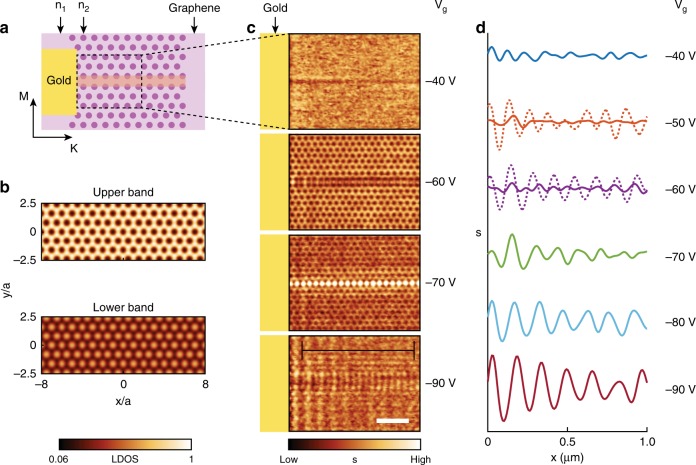
Gate-tunable plasmonic response of a graphene-based photonic crystal. **a** Schematic of the photonic crystal structure with an engineered domain wall in the middle, highlighted in orange. Color contrast represents carrier density modulation *n*_1,2_ in graphene. A gold antenna in the left serves as a plasmon launcher. The scanned area in panel **c** is marked with a dashed box. K/M directions in BZ are marked with arrows. **b** Simulated LDOS maps for upper and lower plasmonic bands. **c** Experimental near-field images *s*(**r**,*ω*) acquired at different gate voltages at *T* *=* 60 K and laser frequency *ω* = 904 cm^−1^. At *V*_g_ = −40 V, only faint plasmonic fringes are observed. At *V*_g_ = −60 V, a hexagonal pattern of dark spots emerges. At *V*_g_ = −70 V, a 1D domain wall state appears in the middle of the image. At *V*_g_ = −90 V, propagating plasmons are launched by the gold antenna on the left. This latter image also reveals a hexagonal pattern of bright spots. Black solid line marks the location of the line profiles in panel **d**. Scale bar: 400 nm. **d** Line profiles *s*(**r**,*ω*) taken in the photonic crystal region away from the domain wall. Dotted lines display the corresponding line profiles in un-patterned region. The systematic increase of SPP wavelength *λ*_p_ with carrier density *n*_s_ matches the expected scaling: $\lambda _{\mathrm{p}} \propto \frac{{v_{\mathrm{F}}\sqrt {n_{\mathrm{s}}} }}{{{\it{\epsilon }}\omega ^2}}$

**Fig. 3:**
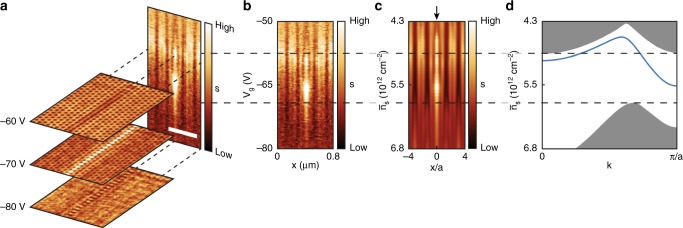
Gate dependence of the 1D plasmonic domain wall state. **a** Stacked near-field images (analogous to Fig. 2c) and detailed voltage-dependent contrast across the artificial domain wall (vertical panel) collected at *ω* = 904 cm^−1^. Scale bar: 400 nm. **b** The same data as panel a vertical panel, displayed in 2D false color map of near-field signal *s* as a function of gate voltages *V*_g_ and position *x* across the domain wall. **c** Simulated voltage-dependent measurement based on point dipole model. $\bar n_{\mathrm{s}}$ is the averaged carrier density. The position of the domain wall is marked by an arrow. The lattice periodicity *a* = 80 nm. **d** 1D plasmonic dispersion simulation for the structure shown in Fig. 2a. Gray region corresponds to 2D plasmonic modes. The blue solid line highlights the 1D domain wall state

A schematic of our tunable photonic crystal is shown in Fig. 1a, with a fully encapsulated graphene monolayer on top of a patterned SiO_2_ gate insulator layer. Due to the presence of the patterned substrate underneath, the graphene carrier density is enhanced at the location of the pillars compared to regions above the voids in SiO_2_ (Fig. 1a inset and Supplementary Fig. 8). To plan our experimental inquiries into SPPs traveling through gate-tunable photonic crystals, we have numerically explored the SPP dispersion for the platform in Fig. 1a (Supplementary Note 6 and Supplementary Figs. 6–8). In the photonic crystal areas, SPPs undergo Bragg reflections due to the periodic pattern of *n*_s_(**r**)^22^, which prompts the formation of a 2D Brillouin zone (BZ) and of plasmonic band structure^27,28^. In the specific case of graphene, the SPP dispersion critically depends on the gate voltage *V*_g_ and the attendant average carrier density $\bar n_{\mathrm{s}}$. Therefore, we augmented the canonical representation of the SPP dispersion in frequency and wave-vector coordinates (*ω* − **k**) into a third dimension given by average carrier density $\bar n_{\mathrm{s}}$ or, equivalently, by the gate voltage *V*_g_ (Fig. 1b). A vertical cut parallel to the *ω* − **k** plane (back panel in Fig. 1b) gives a snapshot of the plasmonic band structure for a specific carrier density $\bar n_{\mathrm{s}}$. At carrier density $\bar n_{\mathrm{s}} = 5.4 \times 10^{12}\;{\mathrm{cm}}^{ - 2}$, our simulations predict the opening of a complete plasmonic bandgap around laser frequency 904 cm^−1^. Provided the carrier density is increased to $\bar n_{\mathrm{s}} = 6.4 \times 10^{12}\;{\mathrm{cm}}^{ - 2}$, the entire plasmonic band structure shifts toward higher frequencies (Supplementary Fig. 7), so that the plasmonic bandgap frequency becomes 920 cm^−1^. A horizontal cut parallel to the $\bar n_{\mathrm{s}} - {\mathbf{k}}$ plane (bottom panel in Fig. 1b) shows the plasmonic wave-vector **k** as a function of carrier density $\bar n_{\mathrm{s}}$ at a given laser frequency *ω* = 904 cm^−1^. In this latter representation, the plasmonic bandgap is evident for $\bar n_{\mathrm{s}} = 4.9 - 5.9 \times 10^{12}\;{\mathrm{cm}}^{ - 2}$. At slightly lower carrier density, e.g. $\bar n_{\mathrm{s}} = 4.5 \times 10^{12}\;{\mathrm{cm}}^{ - 2}$, SPPs reside in the upper plasmonic band but propagating mode is only supported along M direction in the BZ. At slightly higher carrier density, e.g. $\bar n_{\mathrm{s}} = 6.4 \times 10^{12}\;{\mathrm{cm}}^{ - 2}$, the lower band SPPs only propagate along K direction. The $\bar n_{\mathrm{s}} - {\mathbf{k}}$ representation is particularly instructive because experimentally we tune the carrier density to explore the SPP band structure at a fixed IR laser frequency (Figs. 2 and 3).

### Near-field imaging of surface plasmon polaritons

We employed low-temperature near-field optical microscopy to visualize both propagating and localized SPPs in our photonic crystals. In these experiments, a metallic tip of an atomic force microscope (AFM) is illuminated by focused IR radiation from a laser source. The tip acts as an optical antenna which resonantly enhances the electric field at the apex^29^. The tip-scattered light is registered by a detector and the amplitude *s*(**r**,*ω*) and phase *ϕ*(**r**,*ω*) of the corresponding near-field signal are extracted by a proper demodulation scheme (Methods). We analyze the near-field amplitude images *s*(**r**,*ω*) (Figs. 2 and 3) which can be viewed as maps of local electric field *E*_z_ with ~10 nm spatial resolution. We performed the near-field measurements at *T* = 60 K to minimize plasmonic losses due to electron–phonon coupling in the graphene layer, thus obtaining SPPs with ballistic propagation^18^.

Near-field images of SPPs in the photonic crystal are shown in Fig. 2. The scanned area is marked with a dashed box in Fig. 2a. We engineered a domain wall in the middle of this field of view by introducing a shift dislocation of the hexagonal lattice. At gate voltage *V*_g_ = −90 V, we observed SPPs launched by the gold antenna on the left and propagating along the photonic crystal (Fig. 2c) (Supplementary Fig. 5). The SPPs have wavelength *λ*_p_ = 160 nm and falls in the lower plasmonic band in Fig. 1b. Propagating SPPs weaken as the gate voltage is lowered down to *V*_g_ = −70 V, where we approach the bandgap. At even lower gate voltage *V*_g_ = −60 V, we enter the upper plasmonic band. In this latter regime, we observe dark spots forming a hexagonal pattern with the same periodicity as the superlattice. Our analysis below will show that these spots originate from the tip coupling to localized SPPs (Fig. 2b). SPPs are not allowed to propagate along K direction of BZ at *V*_g_ = −60 V. At *V*_g_ = −40 V, faint plasmon fringes are detected (Fig. 2d). The evolution of SPPs with gate voltage in the photonic crystal regions is summarized in Fig. 2d (Supplementary Figs. 1–3). Notably, SPPs amplitude in the photonic crystal region is strongly suppressed at *V*_g_ = −50 V~−70 V, while their propagation is supported in all other gate voltages (Fig. 2d solid line). Un-patterned regions reveal SPPs fringes at all gate voltages (Fig. 2d dotted line). Our observations together with the simulation in Fig. 1b attest to the opening of a complete plasmonic bandgap at *V*_g_ = −60 V~−70 V.

### Plasmonic images and local density of states

The spotted near-field images in Fig. 2c can be interpreted as maps of the localplasmonic density of states (LDOS). Formally, LDOS is defined as the imaginary part of the Green’s function ${\mathrm{LDOS}}\left( {{\mathbf{r}},\omega } \right) = {\mathrm{Im}}\{ G({\mathbf{r}},\omega )\}$^26,30^ and quantifies the response of the system when driven by a dipole source at frequency *ω* and position **r**. The notion of LDOS is commonly employed to describe the various aspects of light-matter interaction including the rate of spontaneous emission^28,31–34^. In regions with enhanced LDOS, the dipole source from a near-field tip couples stronger to SPPs, prompting enhanced near-field signal (Supplementary Fig. 11). At the lowest gate voltage *V*_g_ = −40 V, the SPPs reside away from the plasmonic bandgap, resulting in near-constant LDOS and therefore near uniform nano-infrared signal. At intermediate gate voltages *V*_g_ = −60 V, SPPs occupy the upper plasmonic band with energy proximate to the bandgap (Fig. 1b). Under the latter setting, LDOS at the location of the SiO_2_ dielectric pillars is suppressed (Fig. 2b top panel), leading to dark spots above the dielectric pillars in near-field images (Fig. 2c). At gate voltage *V*_g_ = −70 V, SPPs inhabit inside the plasmonic bandgap but still show lower LDOS on top of the dielectric pillars, due to finite damping rate of graphene SPPs. At the highest attainable gate voltage *V*_g_ = −90 V, SPPs reside in the lower plasmonic band (Fig. 1b). Simulation shows a factor of 16 enhancement of the LDOS at the pillar locations (Fig. 2b bottom panel) which is attested by a bright hexagonal contrast in experimental images (Fig. 2c).

It is worth pointing out that the evolution of the local contrast with gate voltages cannot be explained solely by the variation of local conductivity in graphene. The image taken at *V*_g_ = −40 V (Fig. 2c) is nearly uniform, even though there is still a variation of local conductivity due to the patterned dielectric layer. Likewise, the image taken at *V*_g_ = −90 V (Fig. 2c) shows very weak contrast, even though the variation in conductivity is maximized at the highest gate voltage. The most prominent contrast observed close to the plasmonic bandgap at *V*_g_ = −50 V~−70 V confirms that the nano-infrared contrast in our patterned devices is rooted in the variation of LDOS. This conclusion is further attested by simulations that also link the spotted contrast to the variations in the LDOS (Fig. 2b and Supplementary Figs. 9 and 10).

The observed LDOS enhancement factor of 16 is limited in our structures by plasmonic damping rate and could reach even higher values in plasmonic media with suppressed electron-phonon scattering and enhanced quality factor. At low temperature, our plasmonic devices have a plasmonic damping rate around 10 cm^−1^, which corresponds to quality factor *Q* *=* 100 and SPP propagation length 4 um^18^. When temperature is raised to room temperature, the quality factor decreases down to *Q* *=* 25 due to electron-phonon interaction. Thus, the performance of our devices at ambient is not fully satisfactory. Nevertheless, we wish to point out that the plasmonic/photonic platform proposed in our work is universal and should work with other 2D materials that do not suffer from strong electron-phonon scattering at room temperature.

### Near-field imaging of plasmonic domain walls

Apart from the 2D SPPs discussed above, we also observed a 1D plasmonic mode confined to the domain wall that we introduced in the middle of the field of view in Fig. 2c. These 1D plasmons have not been previously observed in graphene-based structures and are activated when the gate voltage resides within the plasmonic bandgap, at *V*_g_ = −65V for our devices (Supplementary Fig. 2).

The voltage dependence of the 1D plasmons is revealed by scanning the same line across the domain wall while tuning the gate voltage (Fig. 3b). The characteristic bright contrast only appears in a narrow gate range of *V*_g_ = −63 V~−70 V, which matches well with the gate voltage of the plasmonic bandgap (Fig. 2c). Near-field simulations based on the point dipole model detailed in Supplementary Note 6 are displayed in Fig. 3c. Our simulations (Fig. 3c) reproduced the emergence of strong plasmonic response localized to the domain wall at *V*_g_ = −65 V, and dark spots contrast outside the domain wall (Fig. 3b). We repeated the gate dependence measurements at multiple laser frequencies (Supplementary Fig. 4). These latter data along with the simulations of the 1D plasmonic dispersion (Fig. 3d), allow us to attribute the 1D plasmonic mode to the domain wall state residing within the plasmonic bandgap.

## Discussion

The graphene photonic crystal investigated in this work is unique in multiple ways. First, our photonic crystal system is readily tunable through solid state gating effect. Light-matter interaction in the photonic crystal can be turned on and off using gate voltage. We found that the plasmonic LDOS can be varied by as much as 16-fold by tuning the gate voltage across the bandgap. Second, the pristine nature of the graphene is preserved despite close proximity to the patterned superlattice dielectric. Finally, our devices support 1D plasmonic modes confined to an engineered domain wall. Notably, these domain wall states are valley polarized (helical), in the sense that the state from one valley of the plasmonic band propagates only in one direction. Experimental verification of the valley polarization requires a chiral launcher that selectively couples to a single plasmonic valley and is beyond the scope of this study. Provided the photonic crystal breaks inversion symmetry, these domain wall states can become topologically-nontrivial, protected by valley Chern number^27^.

Our gate-imprinted platform paves the way to the design of more elaborate infrared circuits that host valley plasmons^27,35–39^, topological edge states^40–43^ and adds to the toolkit of photonic-crystal-controlled light-matter interactions^44,45^. The top–down fabrication technique for the photonic crystal presented here is fully compatible with the state-of-the-art MOSFET devices, making our system a viable candidate for future electrostatically-reconfigurable plasmonic integrated circuits^46^. Our patterned dielectric arrays also modify the electronic properties of graphene^26^ and the interplay between electronic and plasmonic properties would be of interest to future exploration.

## Methods

### Cryogenic near-field imaging techniques

Cryogenic near-field imaging is accomplished using a home-built scattering-type scanning near-field optical microscope (s-SNOM)^29^. The system is coupled to a continuous-wave CO_2_ laser (Access Laser). The s-SNOM setup is based on a taping mode AFM operating in ultra-high vacuum (UHV) and cryogenic temperatures. The incoming laser beam is focused on the AFM tip using a high-NA off-axis parabolic mirror inside the UHV chamber. The metallic AFM tip is tapped at a frequency of ~250 kHz. A pseudo-heterodyne interferometric detection module is implemented to extract both near-field amplitude *s*(**r**,*ω*) and phase *ϕ*(**r**,*ω*) from the tip-scattered light. The images showed are the near-field amplitude *s*(**r**,*ω*) normalized to the gold contact next to graphene. Thermally evaporated gold serves as a good reference for the detected signal. To properly suppress background contributions to the near-field signal, we demodulate at the third harmonic of the tip tapping frequency.

### Sample fabrication

The hexagonal pillar patterned dielectric superlattices (PDSLs)^26^ were fabricated by plasma etching the SiO_2_ using a thin PMMA mask. Si substrates with thermally grown SiO_2_ of thickness 285 nm were spin coated with a layer of 495 A2 PMMA of thickness 50 nm. The hexagonal pattern was written by e-beam lithography in a Nanobeam nB4 system at a current of 300–400 pA. SiO_2_ pillars were etched in an Oxford Plasmalab 80 Plus system using a mixture of CHF_3_ gas (40 sccm) and Ar gas (5 sccm) to a height of ~50 nm. The PMMA mask was removed through an O_2_ plasma etch and the PDSL cleaned by piranha chemical etching. The resulting superlattice is an array of pillars with 46 nm diameter and 58 nm height that form a hexagonal lattice with a period of 80 nm. A graphene and hexagonal boron nitride heterostructure was placed onto the PDSL by mechanical transfer. The heterostructures consisted of a top h^11^BN of 7 nm, a layer of graphene, and a bottom h^11^BN layer of 4 nm. Metal contacts and launchers were deposited using standard e-beam lithography processes. All the reported back-gate voltages are measured from charge neutrality point of the devices.

The monoisotopic h^11^BN single crystals were synthesized by metal flux method. High purity elemental ^11^B (99.41 at%) was first mixed with Ni and Cr powders in a weight ratio of 1:12:12. These materials were then loaded into an alumina crucible and heated to a molten state with a flowing mixture of N_2_ (95%) and hydrogen (5%) at 1550 °C. The h^11^BN crystals were precipitated on the flux surface by slowly cooling (1 °C/h) to 1500 °C. After the growth process, it was quickly quenched to room temperature. The crystal flakes were obtained from the solidified flux with tape^47^. Optical properties of the h^11^BN single crystals were measured using broadband Fourier-transform infrared spectroscopy method (Supplementary Fig. 6).

## Supplementary information

Supplementary Information

## Data Availability

The data that support the findings of this study are available from the corresponding author upon reasonable request.
