# Supplementary material for: Photonic crystal for graphene plasmons
Source: Nat Commun. 2019 Oct 21;10:4780. doi: 10.1038/s41467-019-12778-2 (PMC6803641; doi:10.1038/s41467-019-12778-2)
Supplement: Supplementary file 1 — Supplementary Information [file 41467_2019_12778_MOESM1_ESM.pdf]

Photonic Crystal for Graphene Plasmons

L. Xiong et al.

This PDF file includes:

Supplementary Note 1-7

Supplementary Figure 1-11

Supplementary References 1-9

Supplementary Note 1: Near-field images at other gate voltages and regions:

Supplementary Figure 1 shows the plasmonic response of the photonic crystal at all gate voltages. The general trends are the same as Figure 2 in the main text. Supplementary Figure 2 shows detailed gate voltage dependence of the domain wall states. It is clear that the domain wall states only exist within a narrow range of gate voltage around $V_g = -70$ V, when probed with laser frequency $\omega = 904\ \mathrm{cm}^{-1}$. Supplementary Figure 3 shows the plasmonic response in un-patterned graphene region.

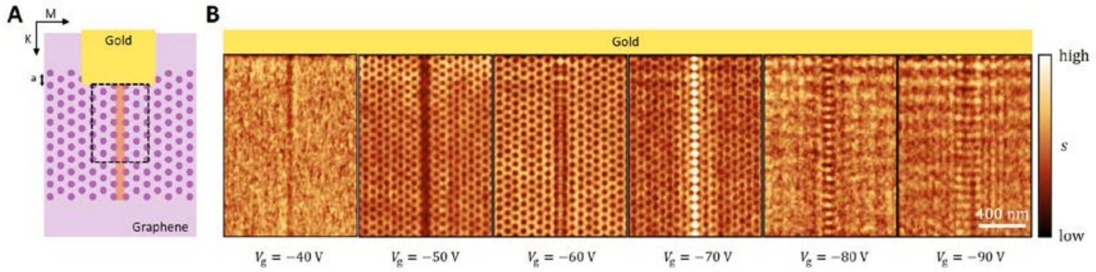

Supplementary Figure 1: **Voltage dependence of the plasmonic response.** (**A**) Schematic of the sample structure with a designed domain wall in the middle. Scan region in panel (**B**) is marked by dashed box. (**B**) Experimental near-field images at different gate voltages at T = 60 K.

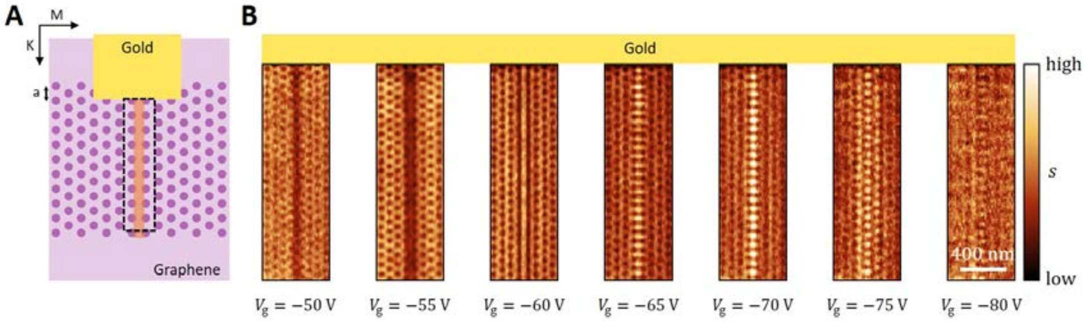

Supplementary Figure 2: **The evolution of the plasmonic domain wall states with gate voltages.** (**A**) Schematic of the sample structure with a designed domain wall in the middle. Scan region in panel (**B**) is marked by dashed box. (**B**) Experimental near-field images near the artificial domain wall at different gate voltages at T = 60 K.

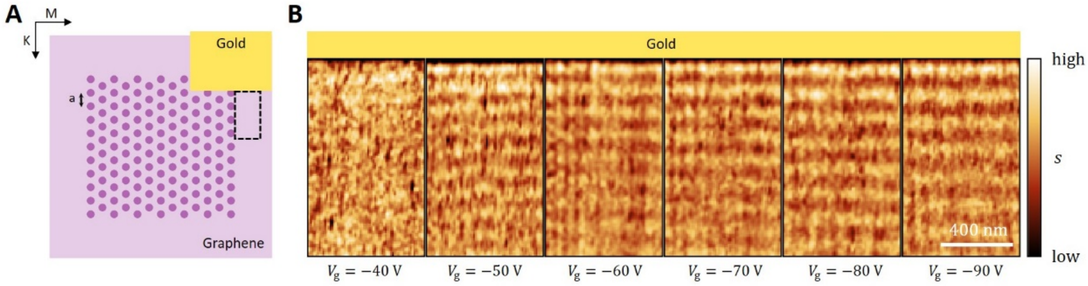

Supplementary Figure 3: **Plasmonic response in un-patterned graphene region.** (**A**)

Schematic of the sample structure. Scan region in panel (**B**) is marked by dashed box. (**B**) Experimental near-field images at different gate voltages at T = 60 K.

Supplementary Note 2: Frequency dependence of the domain wall state:

Supplementary Figure 4 shows the plasmonic domain wall state probed at various laser frequencies. Supplementary Figure 4A shows the near-field image around the artificial domain wall while Supplementary Figure 4B shows gate sweep measurement across the domain wall, all measured at laser frequency $\omega = 888\ \mathrm{cm}^{-1}$. Supplementary Figure 4C, D measure the same physical quantities at laser frequency $\omega = 905\ \mathrm{cm}^{-1}$. Because plasmon wavelength scale as $\lambda_\mathrm{p} \propto \frac{v_\mathrm{F}\sqrt{n_\mathrm{s}}}{\epsilon\omega^2}$, in order to keep the wavelength constant, a 2% increase of laser frequency ω has to be compensated by 8% increase of carrier density n_s, or equivalently gate voltage V_g. In Supplementary Figure 4B, the domain wall state appears at gate voltage $V_\mathrm{g} = -56\ \mathrm{V} \sim -62\ \mathrm{V}$. While in Supplementary Figure 4D, the domain wall state appears at gate voltage $V_\mathrm{g} = -62\ \mathrm{V} \sim -70\ \mathrm{V}$, which is a ~10% increase in gate voltage. This unequivocally proves that we are indeed probing the plasmonic domain wall state which is tightly confined at the artificial domain wall and resides in the plasmonic bandgap.

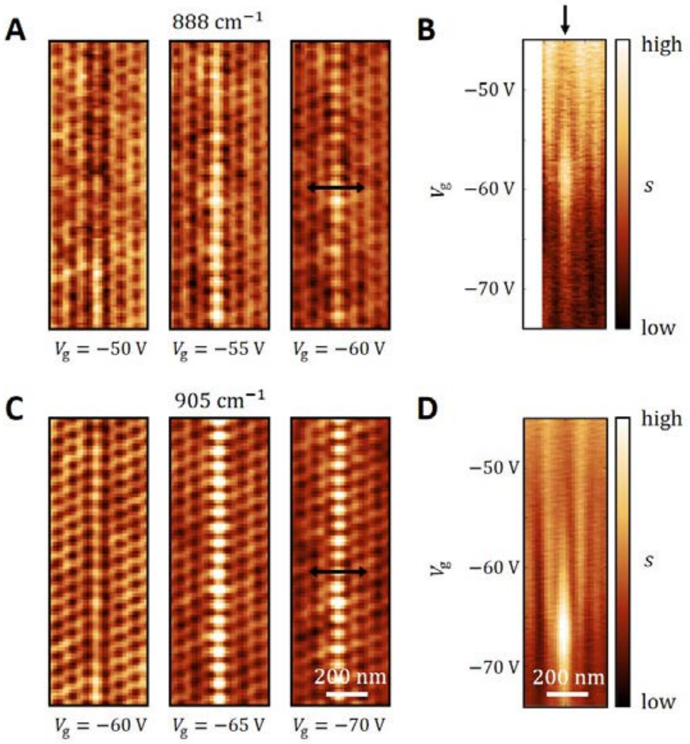

Supplementary Figure 4: **Frequency dependence of the plasmonic domain wall state.** (**A, B**) Near-field imaging (**A**) and gate sweep measurement (**B**) at laser frequency $\omega = 888\ \mathrm{cm}^{-1}$. Black arrow (panel (**A**)) indicates scan position in panel (**B**). Location of the artificial domain wall is marked by black arrow in panel (**B**). (**C, D**) Same

measurement as (**A, B**) with laser frequency $\omega = 905\ \mathrm{cm}^{-1}$. All measurements are taken at $\mathrm{T} = 60\ \mathrm{K}$.

Supplementary Note 3: Gold-launched SPPs:

The high gate voltage $V_\mathrm{g} = -90\ \mathrm{V}$ image in Figure 2c shows gold-launched propagating SPPs in the photonic crystal [1]. The λ_p wavelength fringes are generated due to the interference of laser beam #1 and #2 in Supplementary Figure 5. The incoming laser beam simultaneously reaches the tip (beam #1) and gold launcher (beam #2). Laser beam #1 is back-scattered directly by the tip and registered on the detector. Laser beam #2 first reaches gold launcher which launches SPPs of wavelength λ_p. The SPPs travel for a distance x until it reaches the tip and scattered out for detection. The two beams interfere on the detector with a phase difference of $\Delta\phi = \frac{2\pi}{\lambda_\mathrm{p}}x$, due to propagation phase delay in graphene. The phase delay caused by light propagating in air can be neglected as $\lambda_\mathrm{p} \ll \lambda_0$, where λ_0 is the free-space laser wavelength. This creates the observed oscillatory fringes in the near-field image with λ_p wavelength.

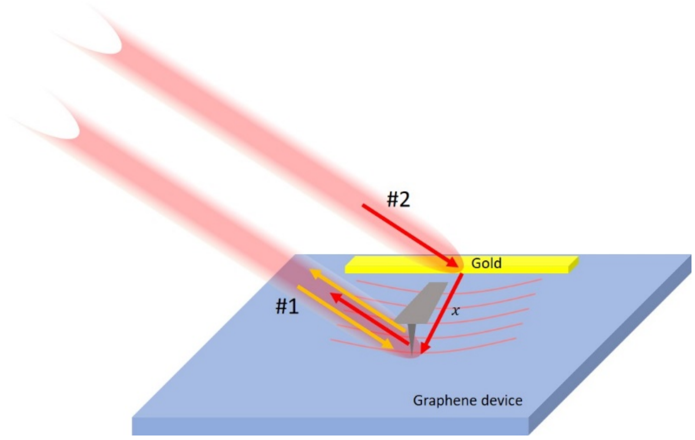

Supplementary Figure 5: **Detection scheme for gold-launched SPPs.** The detected optical signal is the interference between beam #1 and #2. Beam #1 is the direct scattering from the tip. Beam #2 first reaches gold antenna and propagate in the graphene device as SPPs and finally scatter out into far field light at the location of the tip.

Supplementary Note 4: Tip-launched SPPs:

The intermediate gate voltage $V_\mathrm{g} = -50 \sim -70\ \mathrm{V}$ images in Figure 2c show hexagonal pattern of dark spots. This hexagonal pattern originates from coupling of the tip dipole source and the localized plasmonic mode in the photonic crystal. The $E_\mathrm{z}(\mathbf{r})$

pattern launched by a point source is described by the retarded real-space Green's function at the excitation frequency ω,

$$G(\mathbf{r},\mathbf{r}';\omega) = \sum_n \frac{\psi_n(\mathbf{r})\psi_n^*(\mathbf{r}')}{\omega_n - \omega + i0} = P\sum_n \frac{\psi_n(\mathbf{r})\psi_n^*(\mathbf{r}')}{\omega_n - \omega} + i\pi \sum_n \psi_n(\mathbf{r})\psi_n^*(\mathbf{r}')\delta(\omega_n - \omega) \tag{1}$$

Here $\psi_n(\mathbf{r})$ is the plasmonic eigen-mode wavefunction, ω_n is its eigen-frequency, the sum runs over all possible plasmonic states, and P denotes the Cauchy principle value. The scattered near-field signal $s(\mathbf{r},\omega)$ is related to E_z directly under the tip by

$$s(\mathbf{r},\omega) \sim G(\mathbf{r},\mathbf{r};\omega) \propto \mathrm{LDOS}(\mathbf{r},\omega) \tag{2}$$

Where $\mathrm{LDOS}(\mathbf{r},\omega)$ is the local plasmonic density of states at position $\mathbf{r}$ and frequency ω. Thus, the near-field image represents a local map for LDOS.

A more realistic treatment is to model the tip as a point dipole source with the effective separation a = 30 nm (tip radius) from the sample surface. The corresponding Green's function is given by [2]

$$G_{\mathrm{tip}}(\mathbf{r},\mathbf{r};\omega) = \int \frac{d^2k}{(2\pi)^2} F(\mathbf{k}) \sum_i \tilde{G}_{\mathbf{k},\mathbf{k}+\mathbf{G}_i}(\omega) e^{-i\mathbf{q}_i\mathbf{r}} F(\mathbf{k}+\mathbf{G}_i)\,, \tag{3}$$

where

$$F(\mathbf{k}) = |\mathbf{k}|^2 e^{-2|\mathbf{k}|a} \tag{4}$$

is a form-factor. Accordingly, the scattered near-field signal $s(\mathbf{r},\omega)$ is a twice-repeated convolution of $G(\mathbf{r},\mathbf{r}';\omega)$ with the function $F(\mathbf{r}) = \int d^3k\, e^{i\mathbf{k}\mathbf{r}} F(\mathbf{k})$, the Fourier transform of $F(\mathbf{k})$. If the spatial variation of the plasmonic Green's function is slow compared to the tip radius, then the scattered near-field signal should still contain the local information about LDOS. (For more sophisticated models that go beyond the point-dipole approximation, see ref. [2])

Supplementary Note 5: Optical constants for h^{11}BN

The infrared dielectric function for isotopically enriched hBN has been reported to have exceptionally narrow phonon line width already at room temperature [3]. To extract the dielectric function of the isotopically enriched hBN used in this study, we performed broadband FTIR measurement and fit the reflectance spectra using a multilayer model consisting of hBN micro-flakes, SiO_2 and Si substrate [4]. The dielectric function of amorphous SiO_2 and Si substrate is separately measured and can be described with multiple Lorentzians, in accordance with previous studies [1]. The dielectric function of the hBN is parameterized with the standard "TOLO" form for polar materials [3]:

$$\varepsilon(\omega) = \varepsilon_\infty \left(\frac{\omega_{LO}^2 - \omega^2 - i\Gamma\omega}{\omega_{TO}^2 - \omega^2 - i\Gamma\omega} \right) \tag{5}$$

where ε_∞, ω, ω_{TO}, ω_{LO} and Γ is the high-frequency permittivity, frequency, the TO and LO phonon frequency and the phonon damping.

The infrared reflection spectra covering both the upper (UR) and lower

Reststrahlen (LR) bands of hBN micro-flakes on SiO_2/Si substrate were collected using a FTIR microscope (Bruker LUMOS). We have performed the measurement both at room temperature (T = 300 K) and low temperature (T = 60 K), see Supplementary Figure 6. In Supplementary Figure 6 the black squares are experimental data and the red dashed lines are the fitting. The fitting parameters for hBN are listed in the figure. In the reflectance spectra, the broad peak near 1080 cm^{-1} is related to the SiO_2 phonons and the sharper peak near 1360 cm^{-1} is due to hBN in-plane phonon. From the room temperature data (Supplementary Figure 6 left panel) we can confirm that the hBN understudy is of isotope-enriched ^{11}B by comparing both the phonon frequency and phonon damping with previous reports [3]. Remarkably, the narrow line-width for h^{11}BN gives rise to almost total reflection (R = 1) at ω_{TO}, much higher than that of natural hBN (R ~ 0.55 at ω_{TO} [1]). With decreasing temperature, the phonon damping is further reduced and the phonon position blue-shifted slightly, also consistent with previous results.

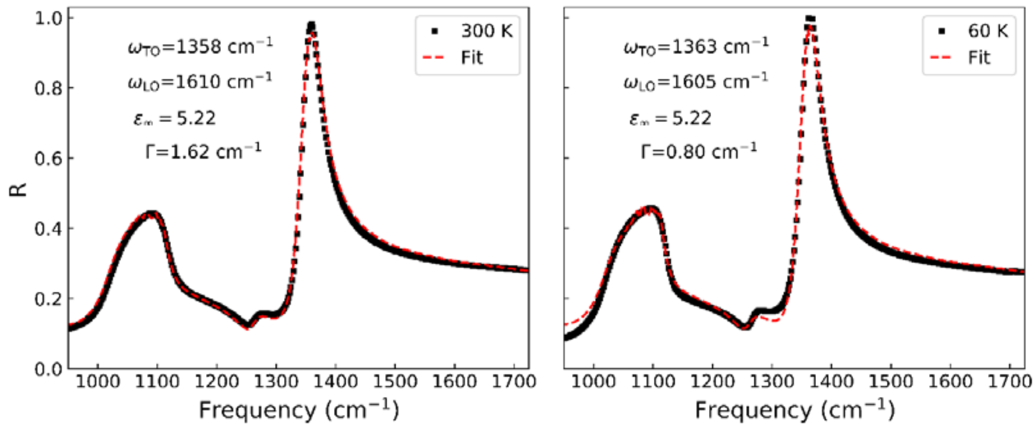

Supplementary Figure 6: **Reflectance spectra of the hBN/SiO_2/Si heterostructure at T = 300 K (left) and T = 60 K (right).** In both panels the black squares are experimental data and red dashed lines are fitting using Lorentzian oscillator model. Fitting parameters related to the isotopically enriched hBN is listed as insets.

Supplementary Note 6: Band structure simulation method:

In this section, we elaborate in detail on the numerical method that is used to calculate the plasmonic band structures. The plasmonic excitation is described by the coupled integral equations of the electric potential $\phi(\omega, \mathbf{q})$ and the carrier density $n(\omega, \mathbf{q})$:

$$\begin{cases} \phi(\omega, \mathbf{q}) = \phi_{ext}(\omega, \mathbf{q}) + \sum_{\mathbf{q}'} \tilde{v}(\omega, \mathbf{q}, \mathbf{q}') n(\omega, \mathbf{q}') \\ n(\omega, \mathbf{q}) = \sum_{\mathbf{q}'} \tilde{\chi}(\omega, \mathbf{q}, \mathbf{q}') \phi(\omega, \mathbf{q}') \end{cases} \quad (6)$$

,where $\tilde{v}(\omega, \mathbf{q}, \mathbf{q}')$ is the effective 2D screened Coulomb interaction, $\tilde{\chi}(\omega, \mathbf{q}, \mathbf{q}')$ is the density response function of graphene, and $\phi_{ext}(\omega, \mathbf{q})$ is the external drive potential [5]. If we use the other set of physical quantities, capacitance and conductivity,

the equivalent description is given as:

$$\begin{cases} \sum_{\mathbf{q}'} \tilde{C}(\omega, \mathbf{q}, \mathbf{q}')\big(\phi(\omega, \mathbf{q}') - \phi_{ext}(\omega, \mathbf{q}')\big) = e^2 n(\omega, \mathbf{q}) \\ n(\omega, \mathbf{q}) = \sum_{\mathbf{q}'} \frac{\mathbf{q}\cdot\mathbf{q}'}{ie^2\omega} \tilde{\sigma}(\omega, \mathbf{q}, \mathbf{q}')\phi(\omega, \mathbf{q}') \end{cases} \tag{7}$$

Here, we used the basic constitutive relations and the continuity equation to derive $\sum_{\mathbf{q}''} \tilde{C}(\omega, \mathbf{q}, \mathbf{q}'')\tilde{v}(\omega, \mathbf{q}'', \mathbf{q}') = e^2\delta_{\mathbf{q},\mathbf{q}'}$ and $\tilde{\chi}(\omega, \mathbf{q}, \mathbf{q}') = \frac{\mathbf{q}\cdot\mathbf{q}'}{ie^2\omega}\tilde{\sigma}(\omega, \mathbf{q}, \mathbf{q}')$.

Combining the above two equations and eliminating $n(\omega, \mathbf{q})$ by substitution yields:

$$\sum_{\mathbf{q}'} \left(\omega\tilde{C}(\omega, \mathbf{q}, \mathbf{q}') + i(\mathbf{q}\cdot\mathbf{q}')\tilde{\sigma}(\omega, \mathbf{q}, \mathbf{q}')\right)\phi(\omega, \mathbf{q}') = \omega \sum_{\mathbf{q}'} \tilde{C}(\omega, \mathbf{q}, \mathbf{q}')\phi_{ext}(\omega, \mathbf{q}') \tag{8}$$

The plasmonic oscillations are self-sustained mode satisfying $\phi_{ext}(\omega, \mathbf{q}) = 0$. From this point and below, we will use $\tilde{\chi}$ instead of $\tilde{\sigma}$ for compact description of plasmonic oscillations:

$$\sum_{\mathbf{q}'} \left(\tilde{C}(\omega, \mathbf{q}, \mathbf{q}') - e^2\tilde{\chi}(\omega, \mathbf{q}, \mathbf{q}')\right)\phi(\omega, \mathbf{q}') = 0 \tag{9}$$

For 2D periodic system, these quantities are evaluated only over a discrete set of momenta, $\mathbf{q} = \mathbf{k} + \boldsymbol{G}$, for Bloch states with a Bloch momentum $\mathbf{k}$ ($\boldsymbol{G}$ is the set of reciprocal lattice vectors). Then, the self-oscillation condition can be casted into a matrix nonlinear eigenvalue problem:

$$\left([\tilde{C}(\omega)] - e^2[\tilde{\chi}(\omega)]\right)|\phi(\omega)\rangle = 0 \tag{10}$$

,where $[\tilde{C}(\omega)]_{\mathbf{q},\mathbf{q}'} = \tilde{C}(\omega, \mathbf{q}, \mathbf{q}')$, $[\tilde{\chi}(\omega)]_{\mathbf{q},\mathbf{q}'} = \tilde{\chi}(\omega, \mathbf{q}, \mathbf{q}')$ and $|\phi(\omega)\rangle_{\mathbf{q}} = \phi(\omega, \mathbf{q})$. The frequency dependence of the dynamic capacitance $\tilde{C}$ originates from the permittivity dispersion of the surrounding materials. For un-patterned SiO_2 layer, both $[\tilde{C}]$ and $[\tilde{\chi}]$ would be diagonal matrices: $\tilde{C}(\omega, \mathbf{q}, \mathbf{q}') = \tilde{C}(\omega, \mathbf{q})\delta_{\mathbf{q},\mathbf{q}'}$, $\tilde{\chi}(\omega, \mathbf{q}, \mathbf{q}') = \tilde{\chi}(\omega, \mathbf{q})\delta_{\mathbf{q},\mathbf{q}'}$.

Now, we briefly explain how the calculation of $\tilde{C}(\omega, \mathbf{q}, \mathbf{q}')$ is done. For a Bloch state with a Bloch momentum $\mathbf{k}$, the ansatz for the solution $\phi(\mathbf{r}, z)$ is given as

$$\phi(\mathbf{r}, z) = \begin{cases} \sum_{\mathbf{q}=\mathbf{k}+\{\mathbf{G}\}} e^{i\mathbf{q}\cdot\mathbf{r}} \times |\phi_{air}\rangle_{\mathbf{q}} e^{-|\mathbf{q}|(z-h_t)} & (z > h_t, \text{Air above top hBN}) \\ \sum_{\mathbf{q}=\mathbf{k}+\{\mathbf{G}\}} e^{i\mathbf{q}\cdot\mathbf{r}} \times \left(|\phi\rangle_{\mathbf{q}} \cosh(\eta|\mathbf{q}|z) - |n_t\rangle_{\mathbf{q}} \sinh(\eta|\mathbf{q}|z)\right) & (0 < z < h_t, \text{Top hBN}) \\ \sum_{\mathbf{q}=\mathbf{k}+\{\mathbf{G}\}} e^{i\mathbf{q}\cdot\mathbf{r}} \times \left(|\phi\rangle_{\mathbf{q}} \cosh(\eta|\mathbf{q}|z) + |n_b\rangle_{\mathbf{q}} \sinh(\eta|\mathbf{q}|z)\right) & (-h_b < z < 0, \text{Bottom hBN}) \\ \sum_{n} |\phi_{PDS}^{\mathbf{k}}\rangle_n T_n^{\mathbf{k}}(\mathbf{r}) e^{\lambda_n^{\mathbf{k}}(z+h_b)} & (z < -h_b, \text{SiO}_2 \text{ Patterned Dielectric Superlattice}) \end{cases} \tag{11}$$

, where $\eta = \sqrt{\epsilon_{\parallel}/\epsilon_{\perp}}$ is the confinement factor of the hyperbolic hBN layers, $\mathbf{r} = (x, y)$, and the graphene is located at $z = 0$. This ansatz is obtained by requiring $\boldsymbol{\nabla} \cdot \mathbf{D} = \boldsymbol{\nabla} \cdot (\boldsymbol{\epsilon}\mathbf{E}) = -\boldsymbol{\nabla} \cdot (\boldsymbol{\epsilon}\boldsymbol{\nabla}\phi) = 0$, thereby assuming quasi-electrostatic limit. For example, in the patterned SiO_2 superlattice layer, $T_n^{\mathbf{k}}(\mathbf{r})$ and $\lambda_n^{\mathbf{k}}$ satisfy

$$\boldsymbol{\nabla}_{\mathbf{r}} \cdot \left(\epsilon(\mathbf{r})\boldsymbol{\nabla}_{\mathbf{r}} T_n^{\mathbf{k}}(\mathbf{r})\right) + {\lambda_n^{\mathbf{k}}}^2 \epsilon(\mathbf{r}) T_n^{\mathbf{k}}(\mathbf{r}) = 0 \tag{12}$$

, where $\boldsymbol{\nabla}_{\mathbf{r}}$ is 2D gradient. It is convenient to solve for $T_n^{\mathbf{k}}(\mathbf{r})$ as linear combinations of plain waves: $T_n^{\boldsymbol{k}}(\boldsymbol{r}) = \sum_{\mathbf{q}=\mathbf{k}+\mathbf{G}} [B_{\mathbf{k}}]_{\mathbf{q},n} e^{i\mathbf{q}\cdot\mathbf{r}}$. Here, $[B_{\mathbf{k}}]$ is the basis transformation matrix from the plain waves to the eigen-mode wavefunction in the patterned SiO_2 layer. If we also express the permittivity function as Fourier series, $\epsilon(\mathbf{r}) = \sum_{\mathbf{G}} \mathrm{E}_{\mathbf{G}} e^{i\mathbf{G}\cdot\mathbf{r}}$, we can rewrite the eigen-value problem of $T_n^{\mathbf{k}}$ into a matrix form:

$$[\epsilon(\mathbf{q}\cdot\mathbf{q}')][B_{\mathbf{k}}] = [\epsilon][B_{\mathbf{k}}][\Lambda]^2 \tag{13}$$

, where $[\epsilon(\boldsymbol{q}\cdot\boldsymbol{q}')]_{q,q'} = E_{q-q'}(\boldsymbol{q}\cdot\boldsymbol{q}')$, $[\epsilon]_{q,q'} = E_{q-q'}$, and $[\Lambda]_{n,n'} = \lambda_n \delta_{n,n'}$. In our specific case, $\epsilon(\mathbf{r})$ is $\epsilon_{\mathrm{SiO_2}}$ inside the pillars, and ϵ_{Air} otherwise. Thus, if we set the center of a pillar to be $\mathbf{r} = 0$, the Fourier components of $\epsilon(\mathbf{r})$ are calculated to be $\mathrm{E}_{\mathbf{G}} = \epsilon_{\mathrm{Air}}\delta_{\mathbf{G},\mathbf{0}} + \frac{\pi R^2}{S}\left(\epsilon_{\mathrm{SiO_2}} - \epsilon_{\mathrm{Air}}\right)\frac{2J_1(|\mathbf{G}|R)}{|\mathbf{G}|R}$ (R is the pillar radius, S is the unit cell area, and J_1 is the first order Bessel function of the first kind). Once we obtain $[B_{\mathbf{k}}]$ and $[\Lambda]$ numerically, we match the boundary conditions for each layer: $D_{z,up} = D_{z,down}$ and $\phi_{up} = \phi_{down}$. Then, we can eliminate the set of coefficients $|\phi_{air}\rangle$ and $|\phi_{PDS}^{\mathbf{k}}\rangle$ by substitutions, thereby obtaining the following relations:

$$\begin{cases} [K_{\mathbf{k}}]|n_t\rangle = [K_{\mathbf{k}}](\epsilon_{\mathrm{eff}}[S_{t\mathbf{k}}] + \epsilon_{\mathrm{Air}}[C_{t\mathbf{k}}])(\epsilon_{\mathrm{eff}}[C_{t\mathbf{k}}] + \epsilon_{\mathrm{Air}}[S_{t\mathbf{k}}])^{-1}|\phi\rangle \\ [K_{\mathbf{k}}]|n_b\rangle = ([K_{\mathbf{k}}][C_{b\mathbf{k}}][S_{b\mathbf{k}}]^{-1} - ([C_{b\mathbf{k}}][S_{b\mathbf{k}}][K_{\mathbf{k}}]^{-1} + [S_{b\mathbf{k}}][K_{\mathbf{k}}]^{-1}[Q_{\mathbf{k}}][K_{\mathbf{k}}]^{-1}[S_{b\mathbf{k}}])^{-1})|\phi\rangle \end{cases} \tag{14}$$

, where $\epsilon_{\mathrm{eff}} = \sqrt{\epsilon_{\parallel}\epsilon_{\perp}}$, $[K_{\mathbf{k}}]_{\mathbf{q},\mathbf{q}'} = |\mathbf{q}|\delta_{\mathbf{q},\mathbf{q}'}$, $[S_{t/b\mathbf{k}}]_{\mathbf{q},\mathbf{q}'} = \sinh(\eta|\mathbf{q}|h_{t/b})\,\delta_{\mathbf{q},\mathbf{q}'}$, $[C_{t/b\mathbf{k}}]_{\mathbf{q},\mathbf{q}'} = \cosh(\eta|\mathbf{q}|h_{t/b})\,\delta_{\mathbf{q},\mathbf{q}'}$, and $[Q_{\mathbf{k}}] = [\epsilon][B_{\mathbf{k}}][\Lambda][B_{\mathbf{k}}]^{\dagger}[\epsilon]/\epsilon_{\mathrm{eff}}$. Then, we obtain the matrix expression for the dynamic capacitance $[\tilde{C}]$ using the Gauss' law:

$$|n\rangle = e^2\epsilon_0\epsilon_{\mathrm{eff}}([K_{\mathbf{k}}]|n_t\rangle + [K_{\mathbf{k}}]|n_b\rangle) = e^2[\tilde{C}]|\phi\rangle \tag{15}$$

The final expression for the dynamic capacitance can be found in ref. [6].

The discussions on the density response function $\tilde{\chi}(\omega, \mathbf{q}, \mathbf{q}')$, its semi-phenomenological expression in the case of periodic doping, and the non-local electron responses are well given in the supplementary material of [6]. Here, we simply provide a comparison between the local Drude model and the non-local semi-phenomenological model. For the local Drude model, the density response function is given as:

$$\tilde{\chi}_{\mathrm{Drude}}(\omega, \mathbf{q}, \mathbf{q}') = \frac{\mathbf{q}\cdot\mathbf{q}'}{\pi\hbar^2\omega^2}\tilde{E}_F(\mathbf{q} - \mathbf{q}') \tag{16}$$

, where $\tilde{E}_F(\mathbf{G})$ is the Fourier component of the Fermi-level distribution $E_F(\mathbf{r}) = \sum_{\mathbf{G}} \tilde{E}_F(\mathbf{G}) e^{i\mathbf{G}\cdot\mathbf{r}}$. For the non-local semi-phenomenological model, extra terms follow:

$$\begin{aligned}\tilde{\chi}_{\mathrm{NL}}(\omega,\mathbf{q},\mathbf{q}') = \tilde{\chi}_{\mathrm{Drude}}(\omega,\mathbf{q},\mathbf{q}') + \frac{\mathbf{q}\cdot\mathbf{q}'}{4\pi}\tilde{E}_F^{-1}(\mathbf{q}-\mathbf{q}') \\ + \frac{3v_F^2(\boldsymbol{q}\cdot\mathbf{q}')^2}{4\pi\hbar^2\omega^4}\tilde{E}_F(\mathbf{q}-\mathbf{q}')\end{aligned} \tag{17}$$

The second term is a local correction from the virtual inter-band transition, and the third term is the leading-order non-local correction from the intra-band transition. Supplementary Figure 7 clearly depicts that the non-local effects introduce an overall blue shifts in the band structure. Also, on a closer inspection, the blue shift at **K** point is greater than that at **M** point due to q^4 dependence of the non-local term, which reduces the size of the complete bandgap.

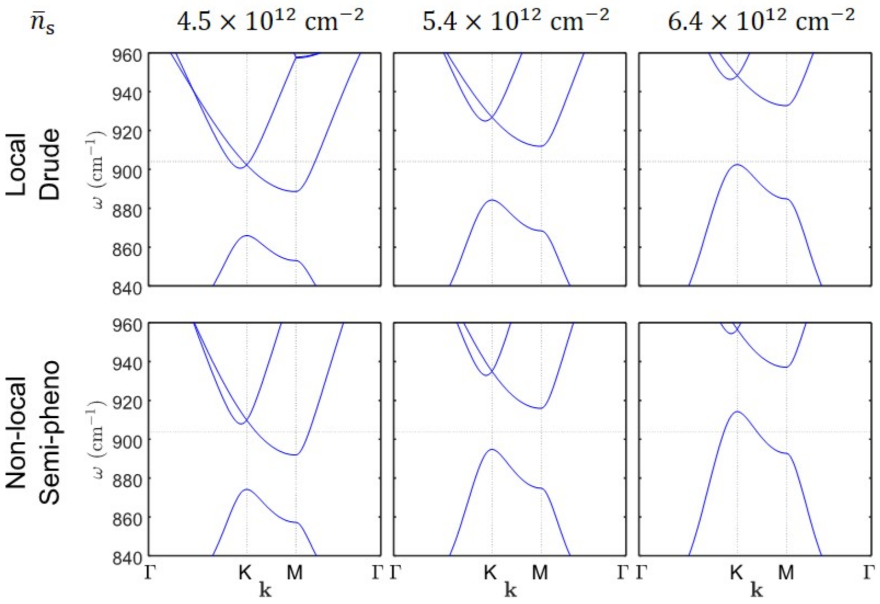

Supplementary Figure 7: **Band structure calculations with and without non-local corrections for the density response function $\tilde{\chi}$.** The top row ignores the non-local corrections, and uses $\tilde{\chi}_{\mathrm{Drude}}$. The bottom row uses $\tilde{\chi}_{\mathrm{NL}}$. The horizontal dotted line refers to $\omega = 904\ \mathrm{cm}^{-1}$.

Lastly, we provide a few comments on the electrostatic simulation of the Fermi-level distribution $E_F(\mathbf{r})$. The local Fermi-level on graphene is given as $E_F = \hbar v_F\sqrt{\pi n_s}$, where n_s is the carrier density. We take the carrier density distribution $n_s(\mathbf{r})$ from electrostatic simulations using COMSOL Multiphysics, see Supplementary Figure 8, and calculate $E_F(\mathbf{r})$ from it. For the values of the fermi velocity v_F, we actually take into account the so-called 'v_F-renormalization', which is a mean-field many-body effect that gives n_s-dependent v_F. We took a phenomenological function of $v_F(n_s)$ from [7], which is based on experimental measurement. v_F-renormalization is also experimentally studied in [8]. We simply assumed that the local Fermi-level

distribution is given as $E_F(\mathbf{r}) = \hbar v_F(n_s)\sqrt{\pi n_s(\mathbf{r})}$.

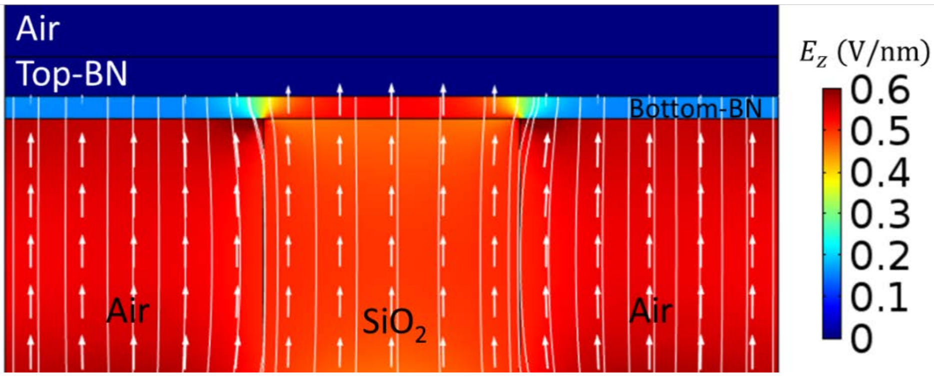

Supplementary Figure 8: **Electrostatic simulation with COMSOL Multiphysics.** The color map represents the z-component of the electric field E_z, the arrow plots the electric field vectors, and the white lines are electric field lines.

Supplementary Note 7: Density of states and near-field signal simulations:

The plasmonic density of states (DOS) or the spectral density/function is defined as

$$\mathrm{DOS}(\omega, \mathbf{k}) \equiv -\Im\left[\sum_n \frac{1}{\omega - \omega_{n,\mathbf{k}}}\right] \tag{18}$$

It is computationally challenging to extract all the eigen-frequencies at every given gate voltage with its dense variation. Thus, we introduce an approximately proper quantity:

$$\mathrm{DOS}(\omega, \mathbf{k}) \sim -\Im\left[tr\left([A]_{\mathbf{q},\mathbf{q}'}\right)\right] \tag{19}$$

, where the response matrix $[A]$ is given as:

$$[A] = \left(\mathbf{1} - e^2\left[\tilde{C}(\omega)\right]^{-1}[\tilde{\chi}(\omega)]\right)^{-1} \tag{20}$$

It is apparent that $[A]$ is not properly defined at the plasmonic resonances, because $[A]^{-1}$ has vanishing eigenvalues. This leads to a diverging trace of $[A]$. Actually, in the limit of vanishing permittivity dispersion and we ignore the nonlocal effect, we have the following exact relation:

$$tr\left([A]_{\mathbf{q},\mathbf{q}'}\right) = \sum_n \frac{1}{1 - \frac{\omega_{n,\mathbf{k}}^2}{\omega^2}} \tag{21}$$

Then, near the vicinity of each resonances ($\omega \sim \omega_{n,\mathbf{k}}$), it is approximated as:

$$tr\left([A]_{\mathbf{q},\mathbf{q}'}\right) \sim \frac{\omega}{2}\sum_n \frac{1}{\omega - \omega_{n,\mathbf{k}}} \tag{22}$$

As we included the permittivity dispersion and the nonlocal effects, the accuracy of this approximation will decrease; however, it is still a good approximation near the

resonances.

In Supplementary Figure 9, we provide the simulation of the total DOS as a function of frequency along with the band structure. The total DOS is defined as

$$\mathrm{DOS_{tot}}(\omega) = \sum_{\mathbf{k}} \mathrm{DOS}(\omega, \mathbf{k}) \tag{23}$$

, where the Block momentum summation is done over the Brillouin zone. As expected, we observe highly suppressed DOS inside the bandgap, and divergent behaviors near the saddle points in the band structure [9]. This result illustrates that our system can be used as a plasmonic platform for tunable Van Hove singularities.

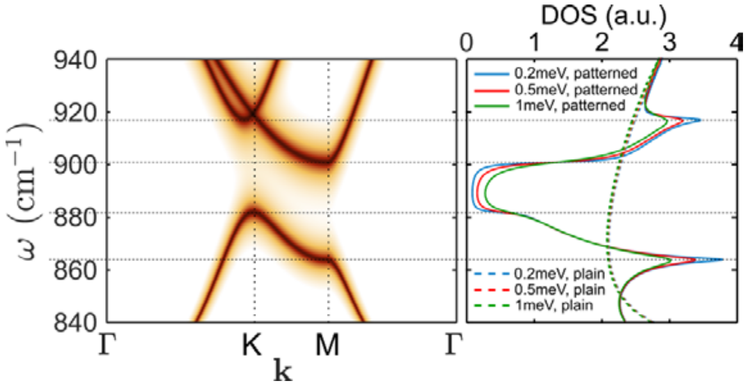

Supplementary Figure 9: **Simulated total DOS of our photonic crystal structure.** Left: Plasmonic band structure at $\bar{n}_s = 4.8 \times 10^{12}\ \mathrm{cm}^{-2}$. Right: Corresponding total DOS evaluated with several choice of Drude losses $\gamma = 0.2, 0.5, 1$ meV.

The plasmonic local density of states (LDOS) is simulated to explain the experimental observation of transition from the bright spots feature below the bandgap to the dark spots features above the bandgap. The LDOS is defined as:

$$\mathrm{LDOS}(\omega, \mathbf{r}) \equiv -\Im\left[\sum_{n,\mathbf{k}} \frac{\left|\mathbf{E}_{n,\mathbf{k}}(\mathbf{r})\right|^2}{\omega - \omega_{n,\mathbf{k}}}\right] \tag{24}$$

, where $\mathbf{E}_{n,\mathbf{k}}(\mathbf{r})$ is the electric field distribution of an eigen-resonance. In Supplementary Figure 10, we calculated LDOS at several frequencies for the structure simulated in Supplementary Figure 9. We provide the square root of LDOS, and the eigen-mode wavefunctions are evaluated at the surface of the top hBN layer. It is clear that LDOS simulations agree with our experimental results that the bright spots feature appears at the lower bands (high gate voltages) and the dark spots feature appears at the upper bands (low gate voltages). The LDOS doesn't vanish inside the bandgap due to the finite loss ($\gamma = 1\ \mathrm{meV} = 8\ \mathrm{cm}^{-1}$) we introduced for the simulation. Examining more carefully the LDOS pattern inside the bandgap, we even find that the residual pattern in the bandgap appears to be the dark spots pattern, which also agrees with the experiments.

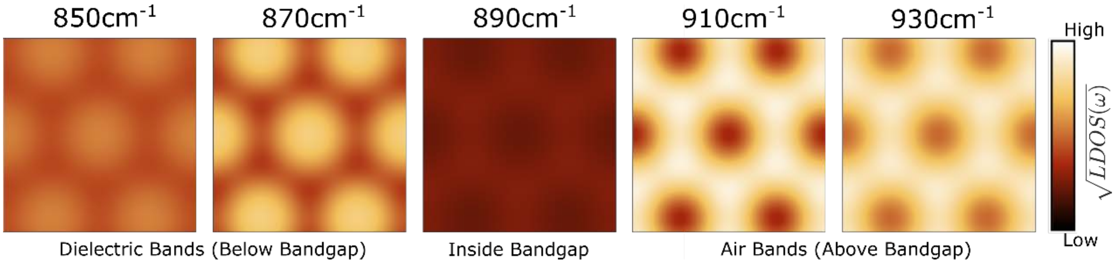

Supplementary Figure 10: **Simulated LDOS of 2D pillar array structure.** LDOS evaluated with several choice of frequencies with a finite loss $\gamma = 1$ meV.

For the simulation of LDOS across the domain wall, it becomes computationally challenging to extract all the eigen-mode wavefunction because there are too many bands (remember that we are solving for a nonlinear eigenproblem, which requires several iterations for each band). Thus, in this case, we simulated the near-field signal directly from the following relation:

$$|\phi_{tot}(\omega)\rangle = [A]|\phi_{excitation}(\omega)\rangle \tag{25}$$

, where $[A]$ is the response matrix introduced earlier, and $|\phi_{excitation}(\omega)\rangle$ is the excitation field. For the excitation field, we assume a vertical dipole right above the surface of the top hBN layer. Then, we define our simulated near-field signal to be the absolute value of the vertical electric field right below the dipole position, and the electric field is calculated from $|\phi_{tot}(\omega)\rangle$. We record this near-field signal by varying the position of the dipole. We may take the norm of the electric field vector instead of taking the vertical component only, which gives pretty much the same results. Supplementary Figure 11 shows the simulated near-field signal in the upper and lower band respectively. They follow exactly the same pattern as the superlattice with low and high near-field signal on top of the pillars, which agrees with the near-field imaging results as well.

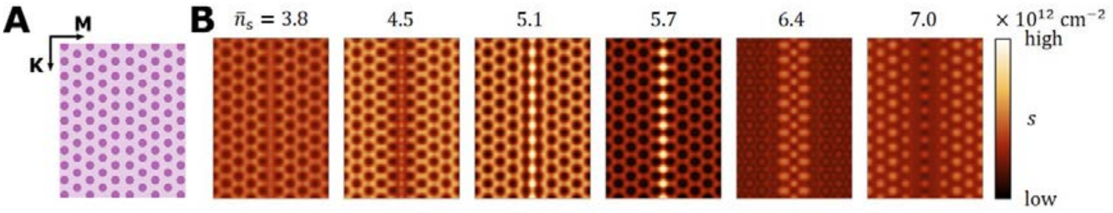

Supplementary Figure 11: **Simulated near-field signals across and around the domain wall.** (**A**) Schematic of the simulation domain with a designed domain wall in the middle. (**B**) Simulated near-field images near the artificial domain wall at different carrier densities.

Supplementary References:

1. Ni, G. X. *et al.* Fundamental limits to graphene plasmonics. *Nature* **557,** 530–533 (2018).
2. Jiang, B.-Y., Zhang, L. M., Castro Neto, A. H., Basov, D. N. & Fogler, M. M. Generalized spectral method for near-field optical microscopy. *J. Appl. Phys* **119,** 54305 (2016).
3. Giles, A. J. *et al.* Ultralow-loss polaritons in isotopically pure boron nitride. *Nat. Mater.* **17,** (2018).
4. Kuzmenko, A. B. Kramers–Kronig constrained variational analysis of optical spectra. *Rev. Sci. Instrum.* **76,** 083108 (2005).
5. Torre, I., Katsnelson, M. I., Diaspro, A., Pellegrini, V. & Polini, M. Lippmann-Schwinger theory for two-dimensional plasmon scattering. *Phys. Rev. B* **96,** 35433 (2017).
6. Jung, M., Fan, Z. & Shvets, G. Midinfrared Plasmonic Valleytronics in Metagate-Tuned Graphene. *Phys. Rev. Lett.* **121,** 086807 (2018).
7. Yu, G. L. *et al.* Interaction phenomena in graphene seen through quantum capacitance. *Proc. Natl. Acad. Sci.* **110,** 3282–3286 (2013).
8. Lundeberg, M. B. *et al.* Tuning quantum nonlocal effects in graphene plasmonics. *Science (80-.).* **357,** 187–191 (2017).
9. Silveiro, I., Manjavacas, A., Thongrattanasiri, S. & García De Abajo, F. J. Plasmonic energy transfer in periodically doped graphene. *New J. Phys.* **15,** (2013).
